# The association between diabetes related medical costs and glycemic control: A retrospective analysis

**DOI:** 10.1186/1478-7547-4-1

**Published:** 2006-01-16

**Authors:** Alan K Oglesby, Kristina Secnik, John Barron, Ibrahim Al-Zakwani, Maureen J Lage

**Affiliations:** 1 Eli Lilly and Company, Indianapolis, IN, USA; 2 Healthcore, Inc., Wilmington, DE, USA; 3 Healthmetrics Outcomes Research, Groton, CT, USA

## Abstract

**Background:**

The objective of this research is to quantify the association between direct medical costs attributable to type 2 diabetes and level of glycemic control.

**Methods:**

A longitudinal analysis using a large health plan administrative database was performed. The index date was defined as the first date of diabetes diagnosis and individuals had to have at least two HbA1c values post index date in order to be included in the analyses. A total of 10,780 individuals were included in the analyses. Individuals were stratified into groups of good (N = 6,069), fair (N = 3,586), and poor (N = 1,125) glycemic control based upon mean HbA1c values across the study period. Differences between HbA1c groups were analyzed using a generalized linear model (GLM), with differences between groups tested by utilizing z-statistics. The analyses allowed a wide range of factors to affect costs.

**Results:**

42.1% of those treated only with oral agents, 66.1% of those treated with oral agents and insulin, and 57.2% of those treated with insulin alone were found to have suboptimal control (defined as fair or poor) throughout the study period (average duration of follow-up was 2.95 years). Results show that direct medical costs attributable to type 2 diabetes were 16% lower for individuals with good glycemic control than for those with fair control ($1,505 vs. $1,801, p < 0.05), and 20% lower for those with good glycemic control than for those with poor control ($1,505 vs. $1,871, p < 0.05). Prescription drug costs were also significantly lower for individuals with good glycemic control compared to those with fair ($377 vs. $465, p < 0.05) or poor control ($377 vs. $423, p < 0.05).

**Conclusion:**

Almost half (44%) of all patients diagnosed with type 2 diabetes are at sub-optimal glycemic control. Evidence from this analysis indicates that the direct medical costs of treating type 2 diabetes are significantly higher for individuals who have fair or poor glycemic control than for those who have good glycemic control. Patients under fair control account for a greater proportion of the cost burden associated with antidiabetic prescription drugs.

## Introduction

The worldwide burden of diabetes is significant and growing so rapidly that it is classified as a global epidemic. The World Health Organization (WHO) estimates that over 177 million individuals live with diabetes, and approximately 4 million deaths each year are related to complications from the disease.[1] While 30 million cases were documented in 1985, 300 million are expected by the year 2025, [1] largely due to the prevalence of type 2 diabetes, which accounts for 90% of all diabetic cases. [2] In the United States, there were approximately 6.5 million cases in 1987 and 12.1 million in 2002. [3,4] Forecasts predict that this number will increase to approximately 14.5 million by 2010 and to 17.4 million by 2020. [5] As the diabetes epidemic expands, associated healthcare costs and demands also continue to increase. [3] For example, the direct medical costs associated with diabetes in the United States in 2002 were estimated to $92 billion. [5] Moreover, the indirect costs associated with lost productivity due to disability and mortality are estimated at an additional $40 billion, resulting in total estimated expenditures for diabetes approaching $132 billion.

Contributing to these large expenditures are the costs associated with diabetes-related complications. Complications associated with diabetes include cardiovascular disease, neuropathy, retinopathy, and nephropathy. [6] The direct medical costs associated with diabetes-related complications totaled $24.6 billion in 2002. [5] These complications substantially increase not only the economic burden for healthcare systems, but also the patient's risk for disability, death, [5] and diminished quality of life. [7-9]

Hemoglobin A1c (HbA_1c_), a clinical measure of ambient blood glucose concentrations over the previous 3 month time period, is recognized as a surrogate measure for the risk of these costly complications. Supporting the use of HbA_1c _as a surrogate measure for complication risk are studies of the U.K. Prospective Diabetes Study (UKPDS) cohort. UKPDS, originally a multi-center clinical trial examining interventions to lower blood glucose and blood pressure among patients with type 2 diabetes, demonstrated that improved glycemic control reduces the risk of microvascular (retinopathy, nephropathy, and neuropathy) and macrovascular (myocardial infarction, stroke) complications. [10] Accordingly, many countries have established guidelines for the treatment of type 2 diabetes that include specific target percentage levels for HbA_1c_. For instance, the American Diabetes Association advocates an HbA_1c _level of less than or equal to 7%. [11] In addition, the National Center for Quality Assurance Health Employer Data and Information Set (NCQA/HEDIS) has established a threshold HbA_1c _value of >9.% to indicate individuals at poor glycemic control when evaluating the performance of managed care plans.[12] Outside of the United States, the United Kingdom's National Institute for Clinical Excellence recommends a target HbA_1c _goal between 6.5% and 7.5%. [13] In addition, the European Diabetes Policy Group identified individuals with HbA_1c _levels of less than or equal to 6.5% as low risk. [14].

As HbA_1c _or glycemic control is considered a surrogate measure for costly diabetic complications, it is also of interest to examine the relationship between HbA_1c _and healthcare utilization and cost. Previous studies have explored the relationship between HbA_1c _levels of patients with type 2 diabetes and healthcare resource use. Whether examining baseline HbA_1c _levels and costs over a 3 year follow up, mean HbA_1c _levels over a 3 year period and adjusted rates for hospital admissions, or the impact of HbA_1c _change on expenditures, the research suggests that successful glycemic control positively affects ensuing healthcare utilization and cost. [15-17] To supplement these findings, the purpose of the present study was to measure the recent healthcare utilization and cost of individuals with type 2 diabetes at varying HbA_1c _levels which correspond to current guidelines. We hypothesized that when considering diabetes-attributable healthcare utilization, lower glycemic control would be significantly associated with lower direct medical costs.

## Methods

Data for this analysis came from the Health Core Managed Care Database. This limited, Health Insurance Portability and Accountability Act (HIPAA) compliant database contains approximately 2.1 million individuals with medical, pharmacy, and eligibility data in the health plan lines of business with complete capture of healthcare provider encounters. Data for the present study were obtained from the Southeastern health plan of the database and covered the time period from October 1, 1998 to April 30, 2003.

We examined the costs associated with a diagnosis of type 2 diabetes by focusing the analysis on individuals with continuous insurance coverage who were diagnosed with type 2 diabetes. Individuals were identified as having type 2 diabetes if they received an oral glucose lowering medication, or both insulin and a diagnosis of type 2 diabetes (ICD-9 of 250.00, 250.10, 250.20, 250.30, 250.40, 250.50, 250.60, 250.70, 250.80, 250.90 (e.g., 250.x0) or 250.02, 250.12, 250.22, 250.32, 250.42, 250.52, 250.62, 250.72, 250.82, 250.92 (e.g. 250.x2)), or at least two diagnoses of type 2 diabetes (ICD-9 of 250.x0 or 250.x2) between the period of October 1, 1998 and April 30, 2001 (e.g. the identification period). We identified an individual's index date as first date of diagnosis of type 2 diabetes or receipt of an antidiabetic agent (a sulphonylurea, amino acid derivative, biguanide, meglitinide, alpha-glucose inhibitor, insulin sensitizing agent, or antidiabetic combination) during the identification period and required that individuals have continuous insurance coverage, at a minimum, from twelve months prior to twenty-four months post-index date. An individual's post-period was allowed to be as long as forty-three months, provided they had continuous insurance coverage during the time-period of interest. We also required that each individual have at least two HbA_1c _values in the post-period. We included in the analyses the 10,780 individuals who fit the above criteria. Individuals were stratified into groups of good (N = 6,069), fair (N = 3,586), and poor (N = 1,125) glycemic control based upon HbA_1c _values of ≤ 7, >7 and ≤ 9, or >9, respectively, where HbA_1c _control groups were based upon the mean HbA_1c _level in the last year of the post-period.

The analysis focused on differences in costs for individuals with different levels of glycemic control. Costs were measured as the direct medical payments associated an inpatient or outpatient claims with a corresponding diagnosis of type 2 diabetes as well as receipt of any outpatient antidiabetic prescription medication. Such payments include both payments by insurance companies as well as payments by patients. All costs were converted into 2003 dollars using the medical care component of the consumer price index (Source: bls.gov). Costs are presented per member per year in order to account for the differences in follow-up periods.

Descriptive statistics included mean (± standard deviation [SD]) and median values for continuous data and relative frequencies for categorical data. Continuous variables were compared with analysis-of-variance (ANOVA) with Scheffe test for multiple-comparisons. This post-hoc test was chosen because it is more stringent (i.e., less likely to make an alpha error) as compared to other tests. Categorical variables were compared based on Pearson chi-squared tests. The length of follow-up was not uniform for all patients.

To determine if costs for post-index events were different between index diagnoses, multivariate generalized linear model techniques were used. Model covariates included demographic characteristics, patient severity, complications and comorbidities of clinical relevance, and HbA_1c _values. Patient demographic characteristics consisted of the individual's age, sex, and type of insurance coverage. Patient severity was proxied by previous resource healthcare use (e.g. prior year costs) as well as a count of distinct medications prescribed during the year prior to index date. In addition, disease prevalence was proxied by an indicator variable equal to one if the individual was diagnosed with type 2 diabetes (ICD-9 of 250.x0 or 250.x2) in the 12 months prior to the index period. Since an individual's index date was based upon first diagnosis of type 2 diabetes or receipt of an antidiabetic agent during the identification period, it is possible to be diagnosed with diabetes prior to the index date. Indicator variables were also constructed for the diabetic complications of nephropathy, retinopathy, foot ulcer, and amputation as well as for the comorbid conditions of myocardial infarction, stroke, coronary artery bypass surgery, and angioplasty. Identification of such variables were dependent upon receipt of a diagnosis or procdure in the one year prior to the index date. The diagnostic and procedure codes used to identify each of the above complications or comorbidities are given in Table 1. Finally, indicator variables were constructed for different rules of identifying individuals with type 2 diabetes. Specifically, indicator variables were set equal to one if an individual received a diagnosis of type 2 diabetes only or if an individual received insulin plus a diagnosis of type 2 diabetes.

**Table 1:** International classification of diseases and current procedural terminology codes for complications and comorbidities

**Complication or Comorbidity**	**International Classification of Diseases – 9 – Clinical Modification Codes**
Nephropathy	39.27, 39.42, 39.43, 39.53, 39.93, 39.94, 39.95, 54.98, 55.4, 55.6, 250.4x, 403.xx, 404.xx, 405.01, 405.11, 405.91, 584.xx, 585.xx, 586.xx, 588.xx, 753.0x, 753.1x, 791.xx, V42, V45.1, V56
Neuropathy	250.6x
Retinopathy	250.5x
Foot Ulcer	707.1x
Amputation	84.1x
Myocardial Infarction	410.0x, 412.x
Stroke/Transient ischemic attack	430.xx – 438.xx
Coronary Artery Bypass Surgery	36.1x, 36.2x, 36.3x
Angioplasty	36.01, 36.02, 36.03, 36.05, 36.09
**Complication or Comorbidity**	**Current Procedural Terminology Codes**
Nephropathy	36800, 36810, 36815, 50300, 50340, 50360, 50365, 50370, 50380, 90920, 90921, 90924, 90925, 90935, 90937, 90945, 90947, 90989, 90993, 90997, 90999
Neuropathy	
Retinopathy	
Foot Ulcer	
Amputation	26910, 27590–27592, 27594, 27596, 27598, 27880–27882, 27884, 27886, 27888, 27889, 28800, 28805, 28810, 28820, 28825
Myocardial Infarction	
Stroke	35301, 35390
Coronary Artery Bypass Surgery	33510–33545, 33572
Angioplasty	92980–92984, 92995–92996

Receipt of any of the diagnostic or procedure codes listed above in the year prior to the index date was used to measure the presence of a complication or comorbidity.

In estimating the multivariate regressions, costs associated with post-index episodes were assumed to follow a gamma-distribution. In addition, costs of care were empirically determined to be linear on the natural logarithm scale. Patient demographic characteristics, indicators of patient severity, type 2 diabetes classification and level of HbA_1c _control were included in the model, while complications and comorbidites covariates with Wald p-values of < 0.15 were considered significant and included in the final model if they did not significantly change the Bayesian Information Criteria scores. The final estimated regression therefore included variables of clinical relevance and best model fit. These models were used to predict estimated costs per member per year with covariates held constant at their sample means. Statistical significance was defined *a priori *at an alpha of less than 0.05. All statistical analyses were performed using STATA version 8.2. [18]

## Results

Table 2 illustrates differences in HbA_1c _levels across different inclusion criteria for the study. Individuals who received oral antidiabetes drugs only during the post-period or were not receiving any antidiabetes medication during the post-period were significantly more likely to have HbA_1c _levels ≤ 7%. In contrast, individuals who received insulin only during the post-period or received an oral antidiabetes drug and insulin during the post-period were significantly more likely to have fair or poor HbA_1c _values. Compared to individuals with fair control, those in the poor control group were more likely to have received both oral antidiabetes drugs and insulin during the post-period and less likely have received only oral antidiabetic medications or no medications during the post-period.

**Table 2:** Identification of individuals included in studyBy HbA_1c _levels

Inclusion Criteria	**Good HbA**_**1c**_	**Fair HbA**_**1c**_	**Poor HbA**_**1c**_
Oral antidiabetic drugs only (no insulin)	4,265 (70%)	2,408 (67%)*	696 (62%)* ^‡^
Oral antidiabetic drugs plus insulin	424 (7%)	562 (16%)*	264 (23%)* ^‡^
Insulin only with ICD-9-CM code of Type 2 Diabetes	346 (6%)	350 (10%)*	112 (10%)*
No diabetic medication with at least two ICD-9-CM diagnoses of Type 2 Diabetes	1,034 (17%)	266 (7%)*	53 (5%)* ^‡^
			
**Sample Size**	**6,069**	**3,586**	**1,125**

Classification at any time during the post-period based upon inclusion/exclusion criteria for study.

In addition, individuals had to be continuously insured from at least 12 months prior and 24 months post index date and have at least 2 HbA_1c _values in the post-period.

Pairwise differences between groups tested using students t-test, z-statistic or Mann-Whitney test.

*Significant below 0.05 compared to the good control group

^‡^Significant below 0.05 between Fair and Poor control groups

Table 3 presents the characteristics of the 10,780 individuals included in the analysis. Among the total population, the average age was approximately 63 years, consisted of more males than females (53% males), and was predominantly Medicare insured (54%). The most common complications or comorbidities were stroke (6.4%) and neuropathy (4.6%). The majority of individuals (56%) maintained good levels of HbA_1c _throughout the study period (e.g., HbA_1c _≤7%).

**Table 3:** Descriptive statistics – By HbA_1c _levels

Variable	**Good HbA**_**1c **_**(≤7)**	**Fair HbA**_**1c **_**(>7 and ≤9)**	**Poor HbA**_**1c **_**(>9)**
**Demographics**			
Mean Age, (SD)	65 (13)	61* (13)	54* ^‡ ^(13)
Sex			
% Female	47%	45%	47%
% Male	53%	55%	53%
Insurance			
% Medicare	62%	47%*	30%* ^‡^
% Commercial	37%	52%*	70%* ^‡^
% Other	1%	1%	<1%
**Patient Severity**			
Mean # of All (diabetic+non-diabetic) Distinct Medications, (SD) prescribed in the pre-period	8.4 (5.5)	8.6 (5.5)	7.8* ^‡ ^(5.6)
Mean total medical costs in 12 month pre-period (SD)	4,524 (8,660)	4,436 (10,774)	3,485* ^‡ ^(7,658)
% Diagnosed with diabetes in the 12 month pre-period.	75%	87%*	88%*
**Complications in Pre-Period**			
% Nephropathy	4.8%	4.4%	3.4%
% Neuropathy	4.1%	5.2%*	5.4%
% Retinopathy	2.6%	3.9%*	3.9%*
% Foot Ulcer	1.6%	1.8%	2.1%
% Amputation	0.2%	0.3%	0.3%
**Comorbidities in Pre-Period**			
% Myocardial Infarction	2.8%	2.9%	2.8%
% Stroke	7.6%	5.1%*	4.5%*
% Coronary Artery Bypass Surgery	0.9%	1.0%	0.6%
% Angioplasty	1.6%	1.2%	1.2%
**Mean Count of HbA**_**1c **_**Tests, (SD)**	5.3 (2.8)	5.6* (2.8)	4.7* ^‡ ^(2.7)
			
**Sample Size**	**6,069**	**3,586**	**1,125**

SD=Standard deviation.

*Significant below 0.05 compared to the good control group.

^‡^Significant below 0.05 between Fair and Poor control groups

Percentages are column percents.

Continuous variables analyzed using OLS regression, Poisson regression or Kruskal-Wallis.

Pairwise differences between groups using student's t-test, z-statistic or Mann-Whitney test.

Table 3 also presents the characteristics of the individuals based upon levels of HbA_1c_. Compared to individuals with good glycemic control, individuals who achieved fair (HbA_1c _values >7% and ≤9%) or poor (HbA_1c _values >9%) glycemic control were significantly younger, were less likely to be Medicare patients, were less likely to be newly diagnosed with diabetes, and were less likely to suffer a stroke. In addition, individuals with good HbA_1c _values were significantly less likely to have been diagnosed with diabetic complications neuropathy or retinopathy. Compared to individuals with good glycemic control, individuals with poor glycemic control had significantly lower prior resource utilization, including total pharmacy medication use, but were more likely to have been diagnosed with retinopathy. There were no differences between the three subgroups with regard to gender or likelihood of having nephropathy, an amputation, foot ulcer, MI, CABG, or angioplasty.

Table 4 presents the results of the multivariate regression analysis for diabetes related prescription drug costs. An examination of the regression reveals that demographic characteristics, patient severity, complications, and type 2 diabetic classification as well as HbA_1c _classification all helped to predict diabetes related prescription drug costs. Specifically, individuals who were older, were diagnosed with nephropathy, or were classified with type 2 diabetes based upon a diagnosis of type 2 or diagnosis of type 2 and receipt of insulin had significantly lower diabetes-related prescription drug costs. In contrast, individuals who were commercially insured or self-insured, as well as those with fair or poor HbA_1c _values had significantly higher total diabetes-related prescription drug costs.

**Table 4:** Cost regressions

**Variable**	**Dependent Variable: Log of Diabetes-Related Prescription Drug Costs**		**Dependent Variable: Log of Diabetes-Related Total Medical Costs**	
	**Coefficient**	**P Value**	**Coefficient**	**P Value**

**Patient Demographics**				
Age	0.994	<0.0001	1.004	0.063
Female^A^	0.978	0.321	0.964	0.329
Commercially Insured^B^	1.742	<0.0001	1.180	0.002
Self-Insured^B^	1.251	0.040	1.432	0.296
**Patient Severity**				
Pre-Period Diabetes-Related Prescription Drug Costs^C^	1.001	<0.0001	---	---
Pre-Period Diabetes-Related Total Medical Costs^C^	---	---	1.000	<0.0001
# of Distinct Antidiabetic Medications Used in Pre-Period	1.136	<0.0001	1.240	<0.0001
**Comorbidities in the Pre-Period**				
Nephropathy	0.902	0.014	1.487	<0.0001
Stroke	---	---	1.317	<0.0001
Foot Ulcer	---	---	1.384	0.0002
Amputation	---	---	1.007	0.981
Retinopathy	1.025	0.520	---	---
**Complications in the Pre-Period**				
Angioplasty	0.902	0.214	---	---
Myocardial Infarction	0.926	0.186	---	---
Coronary Artery Bypass Surgery	0.839	0.055	0.695	0.097
**Type 2 Diabetes Classification**				
Diagnosis of Type 2 Diabetes^D^	0.143	<0.0001	0.515	<0.0001
Diagnosis of Type 2 diabetes and Receipt of Insulin^D^	0.771	<0.0001	1.448	<0.0001
**HbA**_**1c**_**Classification**	---	---		
Fair^E^	1.231	<0.0001	1.196	<0.0001
Poor^E^	1.121	<0.0001	1.243	<0.0001

^A ^– reference category male

^B ^– reference category Medicare insurance

^C ^– pre-period diabetes-related prescription drug costs used only in the diabetes-related prescription drug cost regression and pre-period total diabetes-related total medical costs used only in the total diabetes-related total medical costs regression.

^D ^– reference category individuals who received an oral antidiabetic agent with no diagnosis of type 2 diabetes

^E ^– reference category is classification of good glycemic control

Adjusted for age, gender, LOB (self-insured, commercial, other) prior (1 year) cost, count of distinct medications in the prior (1 year) period, diagnosis of diabetes in one prior (1 year period), type 2 diabetes classification (diagnosis of type 2, diagnosis of type 2 and receipt of insulin, or other), and HbA_1c _classification (fair, poor, or other). Diagnosis of procedure codes of nephropathy, retinopathy, angioplasty, CABG, stroke, MI, amputation, or foot ulcer in the year prior to index date were included in each of the specific models if had Wald p-values of <0.15 and they did not significantly change the Bayesian Information Criteria.

Table 4 also examines the factors which help to predict total diabetes related medical costs. As with the regression for diabetes related prescription drug costs, demographic characteristics, patient severity, complications and comorbidities, type 2 diabetic classification, and HbA_1c _classification all helped to predict costs. Compared to those individuals who received oral antidiabetic agents without a formal diagnoses of type 2 diabetes, individuals who received a diagnoses with no receipt of antidiabetic agents had significantly lower total diabetes related medical costs. In contrast, individuals who received a diagnosis of type 2 diabetes and received insulin had significantly higher total diabetes related medical costs than individuals who received an oral antidiabetic agent only. Commercial insurance, prior medical costs, comorbidities of nephropathy, stroke or foot ulcer were all found to be significant predictors of total diabetes related medical costs. Finally, as with the diabetes related prescription drug regression, individuals classified as having fair or poor HbA_1c _values had significantly higher diabetes related total medical costs than individuals classified as having good HbA_1c _values.

Table 5 examines the estimated costs derived from the multivariate regressions. Specifically, Table 5 presents the estimated mean per member per year costs associated with a diagnosis of diabetes across different HbA_1c _ranges, based upon model covariates held constant at their sample means. Results show that direct medical costs attributable to type 2 diabetes are 16% lower for individuals with good glycemic control than for those with fair ($1,505 vs. $1,801, p < 0.05) control, and 20% lower for those with good glycemic control than for those with poor ($1,505 vs. $1,871, p < 0.05) control. Prescription drug costs were also significantly lower for individuals with good glycemic control compared to those with fair ($377 vs. $465, p < 0.05) or poor ($377 vs. $423, p < 0.05). There were no differences between the fair and poor control groups.

**Table 5:** Estimated mean costs – Per member per year

**Cost**	**Good HbA**_**1c **_**(≤7)**	**Fair HbA**_**1c **_**(>7 and ≤9)**	**Poor HbA**_**1c **_**(>9)**
Diabetic Prescription Drugs	$377 ($366 – $390)	$465* ($450 – $480)	$423* ($400 – $449)
Total Diabetes Attributable Costs	$1,505 ($1,441 – $1,571)	$1,801* ($1,674 – $1,937)	$1,871* ($1,684 – $2078)
**Sample Size**	**6,069**	**3,586**	**1,125**

*Significant below 0.05 compared to the good control group.

No differences between fair and poor control groups.

Confidence intervals given in parentheses.

Estimated costs from a generalized linear model with gamma as the family and logarithmic at the link and all covariates evaluated at the mean.

## Discussion

The findings from this study highlight the significant differences in cost between three levels of glycemic control, namely, good (HbA_1c _≤7%), fair (HbA_1c _>7–9%), and poor (HbA_1c _>9%). These results also offer valuable insight into managed care healthcare resource use by level of HbA_1c _control, specifically antidiabetes medications usage.

Results indicate that level of glycemic control affects the patient's treatment for diabetes when subjects were stratified by use of antidiabetic medication and HbA_1c _level. For example, individuals with good glycemic control were less likely to treat their diabetes with medication and less likely to be prescribed insulin (either alone or in conjunction with oral antidiabetic drugs). By contrast, individuals with poor levels of glycemic control were significantly more likely to treat their diabetes with insulin. This difference most likely exists because the patient in good control is earlier in the course of the disease. [19,20]

Type 2 diabetes is a progressive disease that can initially be effectively managed with diet and exercise in most cases. [19] Eventually, pancreatic beta cell decline coupled with increased insulin resistance eventually results in the need for increased combinations of pharmacotherapy. [21] Patients who are less controlled are more likely to have exhausted available oral treatment options, as these agents require viable insulin production from the pancreas in order to be maximally effective. [22] As beta cell function from the pancreas declines, insulin production will need to be supplemented artificially via insulin injection. [22]

Several studies of administrative claims data have demonstrated an association between HbA_1c _levels and healthcare costs. After examining the relationship between baseline HbA_1c _levels and cost, Gilmer et al. found a $400–$4000 per patient cost savings over a 3 year period for every 1% drop in HbA_1c_.[17] Wagner et al. had similar results after comparing the costs of patients who improved by at least 1% with patients whose HbA_1c _levels did not improve or worsened. Their results indicate that improvements in HbA_1c _levels among patients with diabetes initially assessed as having poor HbA_1c _levels (>10%) were associated with statistically significant lower adjusted healthcare costs within 1 to 2 years of improvement. [16] The results from the present study are in line with this previous research. However, previous studies did not categorize HbA_1c _levels for ease of interpretation by ADA standards or HEDIS guidelines. In the current case, the total diabetes attributable costs were significantly higher for both the fair and poor HbA_1c _groups when compared to patients with good HbA_1c _control (p < 0.05 for both comparisons) (Table 3). Additionally, this trend was evident when examining the prescription drug component of diabetes attributable total costs.

These results point out that even patients considered to have fair glycemic control by HEDIS guidelines, HbA_1c _of >7–9%, have significantly higher total diabetes attributable costs as well as diabetes attributable prescription drug costs, when compared to patients considered to be under good glycemic control, < 7% HbA_1c_. Similarly, in a study by Menzin et al., after controlling for age, sex, cancer, duration of follow-up, and adjusting to 3 years, patients with HbA_1c _levels <8% (good) had a statistically significant reduced probability of inpatient admission compared to those patients in either the 8–10% (fair) or >10% (poor) HbA_1c _category, regardless of long-term diabetic complication. These findings indicate that modest, incremental improvements in HbA_1c _control (improving from poor to fair) may not be adequate for attaining clinically meaningful improvements and suggest that a goal of improving to good HbA_1c _control could result in better patient outcomes and even larger healthcare savings and reduced healthcare resource utilization. It is important to note that this study only examines costs directly attributable to diabetes (e.g. a diagnosis of type 2 diabetes or receipt of an antidiabetic medication) and hence, does not include the costs associated with diabetic complications such as neuropathy or retinopathy that is associated with poor levels of glycemic control. As such, these results may underestimate differences in diabetes related costs for individuals with fair or poor levels of glycemic control.

Interpreting the findings of these analyses must be performed in the context of the study design's limitations. First, the analysis utilized data contained in one health plan located in the Southeastern United States. While this database was deemed to cover an adequately wide geographic distribution, the results may not be generalizable to other populations. Second, while the database includes pharmacological interventions that affect HbA_1c _levels, this analysis was unable to capture patients' exposure to diet and exercise, two interventions shown to have a positive impact on glycemic control. Third, the analysis was unable to control for time from initial diagnosis, a factor that may impact both levels of glycemic control and medical costs. Fourth, the cost analysis was limited to those costs attributable to a diagnosis of diabetes or receipt of an antidiabetic agent. Because this analysis relied on diagnostic codes, the total costs and cost components may therefore be underestimated. Lastly, the reliance on a claims database means that individuals could not be definitively classified as having type 2 diabetes. Specifically, this analysis chose to include individuals who received oral antidiabetic agents independent of a formal diagnosis of type 2 diabetes. However, it should be noted that such a classification is common when conducting retrospective database analyses. [23-25]

## Conclusion

This study yields results supporting the use of glycemic control as a surrogate measure for not only the risk of diabetic complications, but also diabetes attributable healthcare utilization and cost. Results indicate significant differences in the use of diabetic medications between the three levels of HbA_1c _control. In addition, the results indicate that significantly lower diabetes attributable direct medical costs and diabetic prescription drug costs are associated with good HbA_1c _control than with fair or poor HbA_1c _control. These results highlight the fact that there are savings associated with aggressive improvement in patients' glycemic control past the fair category considered acceptable by HEDIS standards. It is anticipated that improved pharmacological therapies will have a significant impact on HbA_1c _levels, and consequently on healthcare costs.

## Competing interests

Funding for this project was provided by Eli Lilly and Company.

## Authors' contributions

AKO and KS conceived the study, participated in the study design, and helped to draft the manuscript. JB and IA participated in the study design, performed statistical analysis and helped to draft the manuscript. MJL participated in the interpretation and results and drafted the manuscript.
